# Embryological and Clinical Outcomes of Oocytes Retrieved from the Pouch of Douglas During Transvaginal Oocyte Pick-Up

**DOI:** 10.3390/jcm15135129

**Published:** 2026-07-01

**Authors:** Selçuk Yetkinel, Gülşen Doğan Durdağ, Didem Alkaş Yağınç, Pınar Çağlar Aytaç, Erhan Şimşek

**Affiliations:** Department of Gynecology and Obstetrics, Adana Dr. Turgut Noyan Application and Research Hospital, Başkent University Faculty of Medicine, 01250 Adana, Turkey

**Keywords:** pouch of Douglas, oocyte pick-up, IVF, intracytoplasmic sperm injection, embryological outcomes, blastocyst development, peritoneal oocytes, assisted reproductive technology, live birth, transvaginal ultrasound-guided oocyte retrieval

## Abstract

**Background/Objectives**: Oocytes retrieved from the pouch of Douglas during transvaginal oocyte pick-up (OPU) have historically been described mainly in isolated case reports and small series. Their developmental competence and clinical relevance remain incompletely characterized. This study aimed to evaluate the embryological and clinical outcomes of oocytes recovered from pouch of Douglas fluid and to compare developmental outcomes between ovarian-derived and pouch of Douglas-derived oocytes. **Methods**: This retrospective observational study was conducted at a tertiary referral university hospital IVF center. Clinical and embryological data collected between February 2023 and June 2025 were retrospectively analyzed. A total of 2423 OPU cycles were screened, and 49 cycles with ≥2 cm of free fluid in the pouch of Douglas before OPU met the inclusion criteria. Following completion of ovarian follicular aspiration, free peritoneal fluid from the pouch of Douglas was aspirated separately and assessed for the presence of oocytes. Cycles were categorized according to whether oocytes were identified in the pouch of Douglas. Baseline cycle characteristics, ovarian response parameters, embryological outcomes, and clinical outcomes were evaluated. Developmental outcomes of ovarian-derived and pouch of Douglas-derived oocytes were compared within the same cycles. **Results**: Oocytes were recovered from pouch of Douglas fluid in 30 of 49 cycles (61.2%). Baseline characteristics, trigger-day hormonal parameters, follicle numbers, and ovarian embryological outcomes were similar between cycles with and without pouch of Douglas oocytes. A total of 44 oocytes were retrieved from pouch of Douglas fluid, of which 36 (81.8%) were metaphase II (MII) oocytes. Twenty-six oocytes demonstrated normal fertilization (2PN), with subsequent cleavage-stage and blastocyst-stage embryo development observed. No significant differences were observed between ovarian-derived and pouch of Douglas-derived oocytes in fertilization or embryo development rates. The proportion of MII oocytes was significantly higher among pouch of Douglas-derived oocytes (81.8% vs. 66.2%, *p* = 0.021). Five embryo transfers involving embryos derived from pouch of Douglas oocytes resulted in clinical pregnancy and live birth, including three transfers exclusively involving pouch of Douglas-derived embryos. No procedure-related complications were observed. **Conclusions**: Oocytes recovered from pouch of Douglas fluid demonstrated fertilization capacity, embryo developmental potential, and the ability to contribute to clinical pregnancy and live birth. These findings suggest that free peritoneal fluid identified before OPU may contain developmentally competent oocytes that would otherwise remain unrecovered. Given the retrospective design, limited number of embryo transfers, and uncertainty regarding the precise origin of these oocytes, the findings should be considered exploratory and require confirmation in larger prospective studies.

## 1. Introduction

Assisted reproductive technologies (ARTs) rely on the retrieval of developmentally competent oocytes during oocyte pick-up (OPU). Despite advances in ultrasound-guided follicular aspiration techniques, premature follicular rupture and inadvertent oocyte loss before or during OPU may still occur, potentially resulting in the presence of free oocytes within the peritoneal cavity, particularly in the pouch of Douglas [1,2].

Historically, oocytes retrieved from the pouch of Douglas have been considered incidental findings during laparoscopy or technically challenging oocyte retrieval procedures. Early studies demonstrated that these oocytes may retain fertilization and cleavage potential comparable to follicular oocytes obtained directly from the ovary [3,4]. Furthermore, isolated reports have documented successful pregnancies and live births following the transfer of embryos derived from oocytes retrieved from the pouch of Douglas [4,5].

More recently, modern ultrasound-guided transvaginal retrieval techniques and advances in embryology laboratory practice have renewed interest in the clinical relevance of peritoneal oocytes. Pereira et al. reported successful fertilization and clinical pregnancy following intracytoplasmic sperm injection (ICSI) of mature oocytes aspirated from free fluid in the posterior cul-de-sac after premature ovulation [5]. Nevertheless, available evidence remains limited to small case series and isolated case reports, and the developmental competence and reproductive potential of oocytes retrieved from the pouch of Douglas remain poorly characterized.

In addition, it remains unclear whether patients in whom oocytes are identified in the pouch of Douglas represent a distinct ovarian response subgroup with different stimulation characteristics, follicular dynamics, or embryological outcomes compared with patients without peritoneal oocytes detected during OPU.

The present study aimed to evaluate the embryological and clinical outcomes of oocytes retrieved from the pouch of Douglas during transvaginal OPU, and to compare demographic characteristics, ovarian stimulation parameters, and clinical outcomes between cycles with and without oocytes identified in the pouch of Douglas.

## 2. Materials and Methods

### 2.1. Study Design and Patient Selection

This retrospective observational study was conducted at a tertiary referral university hospital IVF center. Clinical and embryological data collected between February 2023 and June 2025 were retrospectively analyzed. Clinical follow-up data were collected through 20 May 2026.

During the study period, a total of 2423 transvaginal ultrasound-guided oocyte pick-up (OPU) cycles were screened. Patients with ≥2 cm free fluid identified in the pouch of Douglas before OPU were considered eligible. The ≥2 cm threshold was defined as the maximum vertical depth of the deepest free-fluid pocket measured by transvaginal ultrasonography immediately before oocyte retrieval. Forty-nine cycles (2.0%) met this inclusion criterion, and all eligible cycles were included in the analysis. These 49 cycles were contributed by 47 unique patients; two patients contributed two cycles each.

To minimize the risk of cross-contamination, follicular aspiration of both ovaries was completed before aspiration of fluid from the pouch of Douglas. Following completion of ovarian follicular aspiration, free fluid accumulated in the pouch of Douglas was aspirated separately and immediately assessed by the embryology team for the presence of oocytes.

Cycles were categorized into two groups according to whether oocytes were identified in the aspirated peritoneal fluid from the pouch of Douglas. Given the small number of repeated cycles, all eligible cycles were retained in the analysis.

The study protocol was approved by the Institutional Review Board (Project No: KA26/142; approval date: 10 March 2026) for retrospective analysis of anonymized clinical and embryological data and was conducted in accordance with the principles of the Declaration of Helsinki.

### 2.2. Controlled Ovarian Stimulation and Oocyte Retrieval

Controlled ovarian stimulation (COH) was performed using standard antagonist-based protocols. Gonadotropin dosing was individualized according to patient age, ovarian reserve, and prior response. Follicular development was monitored by serial transvaginal ultrasonography and serum hormone measurements. Final oocyte maturation was triggered using a dual trigger protocol consisting of recombinant human chorionic gonadotropin and a gonadotropin-releasing hormone agonist. Oocyte pick-up (OPU) was performed 36 h after trigger administration under transvaginal ultrasound guidance using a single-lumen aspiration needle (Cook Medical, Bloomington, IN, USA).

Before OPU, the pouch of Douglas was evaluated for the presence of free fluid. In patients with ≥2 cm free fluid identified in the pouch of Douglas, all visible ovarian follicles were aspirated systematically, and particular care was taken to ensure complete ovarian follicular aspiration before aspiration of peritoneal fluid from the pouch of Douglas.

Following completion of ovarian follicular aspiration, the same aspiration needle and tubing were retained. The aspiration needle and line were thoroughly flushed using G-MOPS™ Plus flushing medium (Vitrolife, Gothenburg, Sweden) to minimize carryover of residual follicular fluid or oocytes before pouch of Douglas aspiration. Free fluid accumulated in the pouch of Douglas was subsequently aspirated into a separate collection tube and processed independently by the embryology team. Evaluation for the presence of oocytes was performed in a separate culture dish dedicated to the pouch of Douglas aspirate. Suction pressure was not modified during pouch of Douglas aspiration.

This sequential approach was adopted to minimize the risk of procedural carryover and to reduce the likelihood that oocytes identified in the aspirated peritoneal fluid represented contamination from previously aspirated follicular contents. OPU procedures were performed by experienced reproductive medicine specialists according to the unit’s standard protocol.

### 2.3. Laboratory Procedures and Embryo Assessment

All oocytes were immediately assessed in the embryology laboratory. Oocyte maturity was determined by the presence of the first polar body, and only metaphase II (MII) oocytes were subjected to fertilization procedures. Fertilization was evaluated approximately 16–18 h after ICSI and confirmed by the presence of two pronuclei (2PN).

Embryo development was monitored under standard culture conditions. Embryos were evaluated at the cleavage stage (day 3) and, where applicable, at the blastocyst stage (day 5). Cleavage-stage embryos were assessed based on cell number and fragmentation, while blastocyst-stage embryos were graded according to the Gardner classification system [6]. Embryo grading data were routinely recorded in the laboratory database.

Day 3 embryos were defined as usable cleavage-stage embryos that were transferred or cryopreserved at the cleavage stage and were not subsequently cultured to the blastocyst stage. Day 3 and Day 5 embryo categories were mutually exclusive; therefore, embryos recorded as Day 3 outcomes were not subsequently counted as Day 5 embryos.

For oocytes identified in the pouch of Douglas, all developmental outcomes—including maturation status, fertilization, and subsequent embryo development—were recorded and analyzed separately. All oocytes recovered from pouch of Douglas fluid were identified as cumulus–oocyte complexes at the time of embryological assessment.

### 2.4. Embryo Transfer and Clinical Outcome Definitions

Embryo transfer (ET) was performed according to standard clinical practice, taking into account embryo quality and patient-specific factors.

Transfers involving embryos derived from oocytes retrieved from the pouch of Douglas were identified and categorized as:

- Douglas-only transfers, in which only embryos derived from oocytes retrieved from the pouch of Douglas were transferred.
- Mixed transfers, in which embryos derived from oocytes retrieved from the pouch of Douglas were transferred together with ovarian-derived embryos.

Clinical outcomes were defined as follows:

- Clinical pregnancy: presence of an intrauterine gestational sac with fetal cardiac activity confirmed by transvaginal ultrasound.
- Live birth: delivery of a viable neonate.

Clinical outcomes were followed through delivery, with follow-up data collected through 20 May 2026.

### 2.5. Statistical Analysis

All statistical analyses were performed using SPSS version 26.0 (IBM Corp., Armonk, NY, USA). Analyses were performed at the cycle level. The distribution of continuous variables was assessed visually and analytically. Continuous variables are expressed as a median (interquartile range) due to the non-normal distribution, while categorical variables are presented as a number (percentage).

Baseline cycle characteristics and ovarian response parameters were compared between cycles with and without oocytes identified in the pouch of Douglas. Between-group comparisons were performed using the Mann–Whitney U test for continuous variables and Fisher’s exact test for categorical variables.

Developmental outcomes of oocytes obtained through ovarian follicular aspiration and those retrieved from the pouch of Douglas were compared within cycles in which oocytes were identified in the pouch of Douglas. Developmental rates were calculated separately for ovarian-derived and pouch of Douglas-derived oocytes within each cycle, and paired comparisons were performed using the Wilcoxon signed-rank test.

A two-sided *p*-value < 0.05 was considered statistically significant. Given the exploratory nature of the study and the relatively limited sample size, no correction for multiple comparisons was applied.

## 3. Results

**Figure 1:**
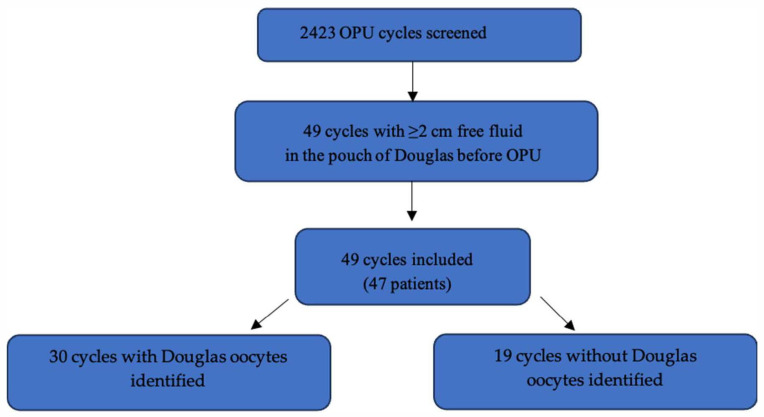
Flow diagram of cycle selection and classification according to the presence of oocytes in pouch of Douglas aspirates.

A total of 2423 OPU cycles were screened during the study period (Figure 1). Among these, 49 cycles (2.0%) met the inclusion criterion of ≥2 cm free fluid in the pouch of Douglas and were included in the analysis. These 49 cycles were contributed by 47 unique patients. Oocytes retrieved from the pouch of Douglas were identified in 30 cycles (61.2%), whereas no oocytes were identified in 19 cycles (38.8%).

### 3.1. Baseline Characteristics and Ovarian Response

Baseline demographic characteristics, hormonal parameters, and ovarian response are summarized in Table 1. There were no statistically significant differences between cycles with and without oocytes identified in the pouch of Douglas in terms of age, ovarian reserve (AMH), trigger-day serum estradiol (E2), luteinizing hormone (LH), or progesterone levels (all *p* > 0.05).

Similarly, no significant differences were observed between the two groups in the number of follicles ≥10 mm or ≥14 mm. Although cycles in which oocytes were identified in the pouch of Douglas showed a trend towards higher numbers of retrieved oocytes, metaphase II (MII) oocytes, and 2PN fertilized oocytes, these differences did not reach statistical significance (Table 1).

No significant differences were observed between the two groups in terms of baseline characteristics or ovarian response.

### 3.2. Comparative Developmental Outcomes of Oocytes Retrieved from Ovarian Follicular Aspiration and the Pouch of Douglas

Comparative developmental outcomes of oocytes retrieved from ovarian follicular aspiration and from the pouch of Douglas are summarized in Table 2. Regarding cycles in which oocytes were identified in the pouch of Douglas, a total of 370 oocytes were obtained through ovarian follicular aspiration and 44 oocytes were retrieved from the pouch of Douglas.

The proportion of mature oocytes was significantly higher among oocytes retrieved from the pouch of Douglas compared with ovarian-derived oocytes (81.8% vs. 66.2%, *p* = 0.021). However, no statistically significant differences were observed between the two groups in terms of normal fertilization (2PN) rates per MII oocyte (72.2% vs. 64.1%, *p* = 0.260), day 3 embryo development rates per 2PN oocyte (34.6% vs. 17.2%, *p* = 0.674), or day 5 blastocyst development rates per 2PN oocyte (38.5% vs. 33.8%, *p* = 0.477).

Similarly, for the overall embryo development rate, defined as the combined number of day 3 and day 5 embryos per 2PN oocyte, no statistically significant difference was observed between oocytes retrieved from ovarian follicular aspiration and those retrieved from the pouch of Douglas (51.0% vs. 73.1%, *p* = 0.116).

### 3.3. Clinical Outcomes Following Embryo Transfer

Clinical outcomes of embryo transfers involving embryos derived from oocytes retrieved from the pouch of Douglas are summarized in Table 3. A total of 5 embryo transfers were performed, all resulting in clinical pregnancy and live birth.

Transfer-level clinical details, including embryo characteristics, gestational age at delivery, and birthweight, are provided in Supplementary Table S1.

Three embryo transfers involved exclusively embryos derived from oocytes retrieved from the pouch of Douglas, all resulting in singleton live births. In two additional cycles, embryos derived from oocytes retrieved from the pouch of Douglas were transferred together with ovarian-derived embryos (mixed transfers). These resulted in one singleton live birth and one dichorionic diamniotic twin live birth.

No procedure-related complications, including bleeding, infection, or other adverse events, were observed following aspiration of peritoneal fluid from the pouch of Douglas.

## 4. Discussion

In this observational study, we evaluated the embryological and clinical outcomes of oocytes retrieved from the pouch of Douglas during transvaginal OPU. The principal finding was that oocytes identified in aspirated peritoneal fluid were not merely incidental laboratory observations, but demonstrated developmental competence, including maturation, fertilization, cleavage-stage and blastocyst development, and successful clinical outcomes after embryo transfer.

The presence of oocytes within the pouch of Douglas has been recognized since the early development of ART. Wikland et al. reported the recovery of a mature oocyte from the pouch of Douglas in a patient who had ovulated before oocyte collection, highlighting that ultrasonography could identify and retrieve oocytes outside the ovarian follicle [2]. Subsequent studies by Dirnfeld et al. and Matson et al. demonstrated that oocytes recovered from the cul-de-sac could fertilize and cleave at rates comparable to follicular oocytes, and could even lead to pregnancy and live birth [3,4]. More recently, Pereira et al. described a clinical pregnancy following ICSI of mature oocytes aspirated from posterior cul-de-sac fluid after premature ovulation [5]. These reports collectively support the biological plausibility of peritoneal oocyte viability.

However, the existing literature remains limited. Most available data consist of early small series or isolated case reports, with limited systematic comparison of stimulation characteristics, embryological progression, and clinical outcomes. In this context, the present study adds important new evidence. To our knowledge, this is among the largest systematically analyzed cohorts evaluating oocytes retrieved from the pouch of Douglas during contemporary transvaginal OPU, and one of the few studies reporting the full developmental sequence from oocyte retrieval to fertilization, embryo development, embryo transfer, clinical pregnancy, and live birth.

A notable finding of our study was that cycles in which oocytes were identified in the pouch of Douglas did not differ significantly from cycles in which no pouch of Douglas oocytes were identified with respect to patient age, AMH level, trigger-day E2, LH and progesterone values, follicle numbers, or oocyte developmental outcomes. This suggests that the presence of oocytes in the pouch of Douglas does not necessarily define a distinct ovarian response profile, but may instead reflect a dynamic event related to follicular rupture, oocyte release, or subtle procedural timing differences within otherwise comparable stimulation cycles.

The mechanisms underlying the presence of oocytes in the pouch of Douglas are likely multifactorial. Premature follicular rupture or premature ovulation before OPU remains the most plausible explanation in some cases, as supported by earlier observations and modern case reports [2,5]. However, mechanical displacement of oocytes during follicular aspiration may also contribute, particularly in cycles with free peritoneal fluid accumulation during retrieval procedures [3]. In addition, physiologic release of oocytes into the peritoneal cavity before tubal capture may represent another possible mechanism. These observations suggest that oocytes identified in the pouch of Douglas may originate through several distinct pathways rather than a single uniform process. Regardless of the underlying mechanism, the clinical importance of maximizing oocyte recovery is well established, as the number of retrieved oocytes is strongly linked to the number of mature oocytes, normally fertilized oocytes, usable embryos, and cumulative reproductive potential [7,8,9]. Therefore, even a small number of additional oocytes retrieved from peritoneal fluid may potentially be clinically relevant, particularly in patients with limited ovarian reserve or borderline oocyte yield.

An important finding of the present study was that developmental outcomes of oocytes retrieved from the pouch of Douglas were broadly comparable to those of ovarian-derived oocytes when evaluated within the same cycles. No significant differences were observed between the two groups in terms of fertilization, cleavage-stage embryo development, or blastocyst development rates. Interestingly, the proportion of MII oocytes was significantly higher among oocytes retrieved from the pouch of Douglas compared with ovarian-derived oocytes. One possible explanation is that mature oocytes may be more likely to separate from the cumulus–oocyte complex and become displaced into the peritoneal cavity during follicular rupture or aspiration. Alternatively, oocytes reaching the peritoneal cavity may represent a subset enriched for mature oocytes. However, these explanations remain speculative. Given the limited sample size and the exploratory nature of the analysis, this finding should be considered hypothesis-generating and warrants further investigation in larger studies.

The embryological findings of the present study further support the developmental potential of oocytes retrieved from the pouch of Douglas. Among 44 oocytes retrieved from the pouch of Douglas, 36 were mature and 26 showed normal 2PN fertilization. Embryo development was observed at both cleavage and blastocyst stages. These findings are consistent with earlier reports suggesting that oocytes recovered from the cul-de-sac may retain fertilization and cleavage potential [3,4]. However, the present study extends previous observations by documenting contemporary embryological outcomes and live births after transfer of embryos obtained from these oocytes.

The clinical outcome data are particularly important. In our cohort, five embryo transfers involving embryos obtained from oocytes retrieved from the pouch of Douglas resulted in clinical pregnancy and live birth. Three of these transfers involved exclusively embryos obtained from oocytes retrieved from the pouch of Douglas, providing supportive clinical evidence that such oocytes can contribute to live birth. In two additional mixed transfers, embryos obtained from oocytes retrieved from the pouch of Douglas were transferred together with embryos obtained from ovarian follicular aspiration; one resulted in singleton live birth and the other in dichorionic diamniotic twin live birth. These findings should be interpreted cautiously because of the limited number of transfers, but they provide supportive clinical evidence for the reproductive potential of oocytes retrieved from the pouch of Douglas.

A major methodological issue in studies of this type is the possibility of contamination from ovarian follicular aspiration. In the present study, this risk was minimized by a strict sequential approach: ovarian follicular aspiration was completed first, and only then was the free fluid in the pouch of Douglas aspirated separately. This procedural sequence is important for interpreting the findings, as it minimizes the likelihood that oocytes identified in the peritoneal aspirate represent carryover from follicular fluid. This point is also consistent with the broader emphasis on standardization, procedural care, and safety in ultrasound-guided OPU [1]. Despite these precautions, the precise origin of the retrieved oocytes cannot be definitively established. Although flushing of the aspiration needle and line, separate collection tubes, and independent embryological assessment were used to minimize procedural carryover, it remains possible that a proportion of oocytes recovered from pouch of Douglas fluid entered the peritoneal cavity during follicular aspiration, follicular collapse, or related procedural events. Therefore, the present findings should be interpreted as evidence of the developmental potential of oocytes recovered from pouch of Douglas fluid rather than definitive proof that these oocytes were present in the pouch before initiation of OPU.

Another relevant finding was procedural safety. No complications, including bleeding, infection, or procedure-related morbidity, were observed after aspiration of peritoneal fluid from the pouch of Douglas. Although the study was not powered to evaluate rare complications, this observation supports the feasibility of the approach when performed carefully and sequentially during routine transvaginal OPU. Potential risks of pouch of Douglas aspiration are expected to be similar to those associated with other transvaginal ultrasound-guided pelvic aspiration procedures and may include bleeding, infection, injury to adjacent pelvic structures, and procedure-related discomfort. However, no such adverse events were observed in the present series. Current good-practice recommendations emphasize that ultrasound-guided OPU is generally safe when performed with appropriate technique and training [1].

This study has limitations. First, although the cohort is large compared with the sparse literature on this topic, the total number of embryo transfers involving embryos obtained from oocytes retrieved from the pouch of Douglas remains small. Second, in mixed embryo transfers involving both pouch of Douglas-derived and ovarian-derived embryos, implantation and live birth outcomes cannot be definitively attributed to a specific embryo source. Third, despite efforts to minimize procedural carryover, the precise origin of oocytes recovered from pouch of Douglas fluid cannot be definitively established. Fourth, the retrospective observational design precludes causal inference regarding why oocytes are present in the pouch of Douglas. In addition, although analyses were performed at the cycle level, the study included 49 cycles contributed by 47 patients, and therefore a small degree of within-patient clustering cannot be excluded. Finally, the findings should be considered exploratory and require confirmation in larger prospective studies.

Despite these limitations, the study provides valuable clinical and embryological information in an area where evidence is scarce. Unlike isolated case reports, this cohort includes systematic comparison between cycle characteristics with and without oocytes retrieved from the pouch of Douglas, detailed embryological follow-up of Douglas-derived oocytes, and clinically meaningful reproductive outcomes. These findings suggest that free fluid identified in the pouch of Douglas before OPU should not be dismissed as an incidental finding. When present, careful aspiration and embryological examination may allow recovery of developmentally competent oocytes capable of contributing to embryo development and live birth.

## 5. Conclusions

Oocytes recovered from pouch of Douglas fluid demonstrated fertilization capacity, embryo developmental potential, and the ability to contribute to clinical pregnancy and live birth. Developmental outcomes of pouch of Douglas-derived oocytes were broadly comparable to those of ovarian-derived oocytes within the same cycles, although the findings should be interpreted in light of the retrospective design and limited number of embryo transfers. These results suggest that free peritoneal fluid identified before OPU may contain developmentally competent oocytes that would otherwise remain unrecovered. Careful aspiration and embryological evaluation of such fluid may allow recovery of additional developmentally competent oocytes in selected cases. Given the uncertainty regarding the precise origin of these oocytes and the exploratory nature of the present findings, larger prospective studies are required to clarify their biological origin, developmental potential, and clinical significance.

## Figures and Tables

**Table 1:** Baseline characteristics and ovarian response according to presence of oocytes retrieved from the pouch of Douglas.

Variable	Douglas Oocyte (−) (*n* = 19)	Douglas Oocyte (+) (*n* = 30)	*p*-Value
Age (years)	34.0 (28.5–39.5)	34.0 (31.0–36.8)	0.673
AMH (ng/mL)	1.16 (0.47–3.24)	2.09 (1.11–3.23)	0.331
Trigger E2 (pg/mL)	635 (287–2026)	1292 (732–2143)	0.170
Trigger LH (IU/L)	2.99 (1.92–3.52)	3.03 (1.80–4.16)	0.715
Trigger progesterone (ng/mL)	0.50 (0.30–1.60)	0.95 (0.53–1.95)	0.140
Follicles ≥10 mm (*n*)	7 (3–15.5)	10 (6–15)	0.475
Follicles ≥14 mm (*n*)	4 (2–11)	6 (3–9)	0.584
OPU oocyte number (*n*)	4 (2.5–11)	8.5 (4.5–17)	0.188
MII oocytes (*n*)	3 (2–7)	5.5 (3–10.8)	0.171
2PN oocytes (*n*)	2 (1.75–4)	4 (3–8)	0.099
Day 3 embryos (*n*)	2 (1–2)	1 (0.75–2)	0.325
Day 5 embryos (*n*)	1 (0.25–3.25)	2 (1–4)	0.384
OPU timing (hours)	36 (36–36)	36 (36–36)	0.676

Values are presented as median (interquartile range) unless otherwise stated. Comparisons were performed using the Mann–Whitney U test for continuous variables and Fisher’s exact test for categorical variables.

**Table 2:** Comparative developmental outcomes of oocytes retrieved from ovarian follicular aspiration and the pouch of Douglas.

Developmental Parameter	Ovarian Follicular Aspiration	Pouch of Douglas Aspiration	*p*-Value
Total retrieved oocytes, *n*	370	44	—
MII oocytes/total oocytes, *n* (%)	245/370 (66.2%)	36/44 (81.8%)	0.021
Normal fertilization (2PN)/MII oocytes, *n* (%)	157/245 (64.1%)	26/36 (72.2%)	0.260
Day 3 embryos/2PN oocytes, *n* (%)	27/157 (17.2%)	9/26 (34.6%)	0.674
Day 5 embryos/2PN oocytes, *n* (%)	53/157 (33.8%)	10/26 (38.5%)	0.477
Total embryos (day 3 + day 5)/2PN oocytes, *n* (%)	80/157 (51.0%)	19/26 (73.1%)	0.116

Data are presented as pooled numbers and percentages. Developmental outcomes of oocytes retrieved from ovarian follicular aspiration and from the pouch of Douglas were compared within cycles in which oocytes were identified in the pouch of Douglas. Statistical comparisons were performed using cycle-level paired developmental rates and the Wilcoxon signed-rank test. MII: metaphase II; 2PN: two pronuclei.

**Table 3:** Embryo transfer outcomes using embryos derived from oocytes retrieved from the pouch of Douglas.

Parameter	Value
Total embryo transfers (*n*)	5
Clinical pregnancies (*n*, %)	5 (100%)
Live births (*n*, %)	5 (100%)
Douglas-only embryo transfers (*n*)	3
Live births after Douglas-only transfer (*n*, %)	3 (100%)
Mixed embryo transfers (*n*)	2
Outcomes after mixed transfer	1 singleton live birth, 1 DCDA twin live birth

Clinical outcomes of embryo transfers involving embryos derived from oocytes retrieved from the pouch of Douglas. Douglas-only transfers refer to cycles in which exclusively embryos originating from oocytes retrieved from the pouch of Douglas were transferred. Mixed transfers refer to cycles in which both Douglas-derived and ovarian-derived embryos were transferred. DCDA: dichorionic diamniotic.

## Data Availability

The data presented in this study are available on reasonable request from the corresponding author. The data are not publicly available due to privacy restrictions.

## Supplementary Materials

The following supporting information can be downloaded at https://www.mdpi.com/article/10.3390/jcm15135129/s1, Supplementary Table S1: Transfer-level clinical outcomes of embryo transfers involving embryos derived from oocytes retrieved from the pouch of Douglas.

## Abbreviations

The following abbreviations are used in this manuscript:

ART	Assisted reproductive technology
COH	Controlled ovarian stimulation
ET	Embryo transfer
ICSI	Intracytoplasmic sperm injection
MII	Metaphase II
OPU	Oocyte pick-up
2PN	Two pronuclei
