# Supplementary material for: Embryological and Clinical Outcomes of Oocytes Retrieved from the Pouch of Douglas During Transvaginal Oocyte Pick-Up
Source: J Clin Med. 2026 Jul 1;15(13):5129. doi: 10.3390/jcm15135129 (PMC13362622; doi:10.3390/jcm15135129)
Supplement: Supplementary file 1 [file jcm-15-05129-s001.zip › jcm-4373396-supplementary.pdf]

Supplementary Table S1. Transfer-level clinical outcomes of embryo transfers involving embryos derived from oocytes retrieved from the pouch of Douglas

**Case**	**Transfer type**	**Embryos transferred**	**Clinical outcome**	**Gestational age at delivery (weeks)**	**Birthweight (gr)**
1	Mixed	Douglas-derived blastocyst (Day 5, 5BB) + ovarian-derived morula	Singleton live birth	39	3920
2	Mixed	Douglas-derived cleavage-stage embryo (Day 3, 8/2) + ovarian-derived cleavage-stage embryo (Day 3, 4/2)	DCDA twin live birth	35	2720 and 2780
3	Douglas-only	Douglas-derived blastocyst (Day 5, 5BB)	Singleton live birth	38	3300
4	Douglas-only	Douglas-derived blastocyst (Day 5, 5BB)	Singleton live birth	35/5	2960
5	Douglas-only	Douglas-derived cleavage-stage embryo (Day 3, 8/1) + Douglas-derived blastocyst (Day 5, 4BC	Singleton live birth	24/2	600
